# Alleviation of iron deficiency in pear by ammonium nitrate and nitric oxide

**DOI:** 10.1186/s12870-022-03826-z

**Published:** 2022-09-12

**Authors:** Jianlong Liu, Jinzhu Wang, Zidong Wang, Min Li, Chenglin Liang, Yingjie Yang, Dingli Li, Ran Wang

**Affiliations:** 1 College of Horticulture, Qingdao Agricultural University, Qingdao, 266109 China; 2 Haidu College, Qingdao Agricultural University, Laiyang, 265200 China

**Keywords:** Ammonium nitrate, Nitric oxide, Pear, Iron deficiency stress, Nitrogen assimilation

## Abstract

**Background:**

Iron is essential for the growth and development of trace elements in plants, and iron deficiency can lead to leaf chlorosis. Ammonium and nitrate are the major forms of nitrogen present in soils. Ammonium nitrate alleviates the chlorosis of leaves caused by iron deficiency, but the mechanism is not clear in pear.

**Results:**

Ammonium nitrate induced the increase of nitric oxide (NO) under iron deficiency. We further analyzed the effect of NO by exogenous NO treatment. The results showed that ammonium nitrate and NO increased the activity of ferric chelate reductase. NO induced the expression of multiple *IRT* genes and promoted the transmembrane transport of irons. Ammonium nitrate and NO promoted the activity of nitrogen assimilation-related enzymes and the nitrogen absorption capacity, and they also increased glutamine synthetase activity. Finally, ammonium nitrate and NO increased chlorophyll synthesis, with subsequent increase in the photosynthetic capacity of plants and accumulation of biomass.

**Conclusion:**

Ammonium nitrate indirectly alleviates the symptoms of plant yellowing by promoting the increase of NO, which increases the response of iron transporters. Both substances increase the nitrogen accumulation in plants. This study demonstrates a new option for minimizing Fe deficiency by regulating the balance between nutrients.

## Background

Iron (Fe) is an essential micronutrient and a universal cofactor that plays a vital role in photosynthesis, respiration, hormone biosynthesis, morphogenetic cellular enzymatic reactions, and the growth and developmental processes of plants [1–3]. Although iron is the element with the fourth-highest abundance in the Earth’s crust, most of it exists in the form of hydroxides with low bioactivity. Fe deficiency is a worldwide horticultural problem, and approximately 30% of the world’s soil is currently Fe deficient, which may trigger chlorosis and reduce fruit yields [4].

Plants utilize two mechanisms to increase Fe uptake capacity to cope with Fe deficiency: strategy I (used by dicots and nongraminaceous monocot plants) and strategy II (used by graminaceous monocots) [5, 6]. Pear, as a strategy I plant, reduces Fe^3+^ to Fe^2+^ by ferric chelate reductase (FCR) in the root surface, and Fe^2+^ then enters the cytoplasm in the root [7]. Before entering the xylem, Fe^2+^ in the cytoplasm is reoxidized to Fe^3+^ and then combined with citric acid and transported aboveground in the xylem, and finally, Fe^3+^ is reduced to Fe^2+^ for assimilation and utilization [8]. Thus, FCR is one of the key enzymes for iron absorption and recycling in plants [9].

Studies have shown that iron deficiency induces various reactions at the root level to increase the uptake of available iron in the rhizosphere. Plants can alleviate iron deficiency stress by increasing iron storage, mainly through three ways: (I) improving the iron reduction ability of root tissue, (II) acidifying the rhizosphere to increase iron solubility, and (III) improving the absorption capacity of root cells to cope with iron deficiency in soil. Ammonium nitrate significantly affected the pH of plant rhizosphere, and then affected the absorption and utilization of iron. Under iron deficiency, the addition of NH_4_^+^ increased the reuse of iron in primary leaves. NH_4_^+^ treatment affected the transfer of iron from primary leaves and stems to young leaves, and significantly increased the iron level in young leaves of maize [10]. In addition, the iron content of spinach shoots significantly increased under the culture of nutrient solution containing NH_4_^+^ [11], further confirming that different forms of nitrogen affect Fe uptake. Fe is also a metal cofactor of enzymes in the nitrogen reductive assimilatory pathway, and iron absorption affects nitrogen assimilation [12]. Nitrate reductase (NR), nitrite reductase (NiR), and glutamate synthase (GOGAT) all require Fe as the Fe-heme group or Fe-S cluster [13].

Nitric oxide (NO) is a signaling molecule that is involved in a variety of physiological processes in response to Fe deficiency [14–19]. Sodium nitroprusside (SNP; NO donor) decreased the pH of soil and increased the available Fe in the soil [20]. In addition, the application of exogenous SNP significantly increased the total iron concentration in leaves by increasing the H^+^-ATPase and Fe^3+^ reductase activities [21]. SNP can also prevent iron deficiency induced oxidative stress, promote iron activation, and regulate the balance of mineral elements [22, 23].

Higher plants produce NO from various enzymatic and non-enzymatic sources [24–27]. Although much progress has been made in the production of NO, there are still many problems to be solved [28]. As a key enzyme in the nitrogen cycle, NR also participates in the biosynthesis of NO [29]. At present, studies have shown that ammonium regulates Fe deficiency responses by enhancing NO signaling in *Arabidopsis thaliana* [30]. However, how ammonium nitrate and NO alleviate the symptoms in pear caused by iron deficiency has not yet been reported.

In this work, we aimed to analyze the role of ammonium nitrate and NO in alleviating iron deficiency in pear. Through the exogenous application of ammonium nitrate and SNP, combined with physiological and biochemical data, the possible regulatory relationship between them was explained. The regulatory mechanisms behind this relationship were also investigated, which elucidated the interaction between ammonium nitrate, NO, and iron deficiency in pear.

## Methods

### Plant material and growth conditions

Pear variety ‘Qingzhen D1’ (*P. communis* L. × *P. bretschneideri* Rehd.) was independently bred at the college of horticulture, Qingdao agricultural university. In vitro shoots of ‘Qingzhen D1’ pear were proliferated on rooting medium that consisted of 1/2 Murashige and Skoog (MS) medium supplemented with 0.2 mg L^− 1^ naphthalene acetic acid (NAA), 1.5 mg L^− 1^ 3-indolebutyric acid (IBA), 3% (w/v) sucrose, and 0.7% (w/v) agar. After 20 days, seedlings with the same growth and development were selected and subjected to the following treatments for 60 days: +Fe (100 μmol L^− 1^ Fe(III)-EDTA), −Fe (1 μmol L^− 1^ Fe(III)-EDTA), −Fe + AN (1 μmol L^− 1^ Fe(III)-EDTA+ 6.85 mmol L^− 1^ NH_4_NO_3_; the application amount of NH_4_NO_3_ was 0.5 times the amount of total nitrogen in NN69 medium), and -Fe + SNP (1 μmol L^− 1^ Fe(III)-EDTA+ 100 μmol L^− 1^ SNP). The NO-elimination experimental treatments were applied for 20 days and consisted of: -Fe (1 μmol L^− 1^ Fe(III)-EDTA), −Fe + cPTIO (1 μmol L^− 1^ Fe(III)-EDTA+ 100 μmol L^− 1^ 2-(4-carboxyphenyl)-4,4,5,5- tetramethylimidazoline-1-oxyl-3-oxide (cPTIO; NO scavenger), −Fe + AN (1 μmol L^− 1^ Fe(III)-EDTA + 6.85 mmol L^− 1^ NH_4_NO_3_), and -Fe + AN+cPTIO (1 μmol L^− 1^ Fe(III)-EDTA + 6.85 mmol L^− 1^ NH_4_NO_3_ + 100 μmol L^− 1^ cPTIO). NN69 medium supplemented with 0.3% (w/v) agar [31], pH 5.8, was used for the cultures, which were grown at 22–27 °C with a 14 h photoperiod, relative humidity (RH) of 65–85%, and light intensity ranges from 6000 to 8000 lx. The samples were immediately frozen in liquid nitrogen and stored at − 80 °C for later use.

### Determination of relative chlorophyll content and photosynthetic characteristics

A portable chlorophyll meter (Konica Minolta SPAD-502, Japan) was used to determine the chlorophyll content, which is expressed as the soil plant analysis development (SPAD) value. The net photosynthetic rate (*Pn*), stomatal conductance (*Gs*), transpiration rate (*Tr*), and intercellular CO_2_ concentration (*Ci*) of the fully expanded leaves, which were the third leaves from the top, were measured using a portable photosynthesis system (PP Systems CIRAS-3, USA). Leaf temperatures were maintained at 25 ± 1 °C. The RH in the assimilation chamber was maintained at 70%, the external CO_2_ concentration remained at 380 ± 10 μmol mol^− 1^, the light intensity was consistent at 200 μmol photons m^− 2^ s^− 1^, the gas flow rate was 300 mL min^− 1^, and the air humidity was 65–85%.

### Analysis of root morphology

Root tip number, root length, root surface area, and average root diameter were scanned (Epson Perfection V800 photo scanner, China), and the relevant data were analyzed with WinRHIZO 2007 software.

### Determination of soluble iron content

A fresh sample was dried and ground into powder, and 1.00 g was placed in a corkscrew test tube. Then, 10 mL of 0.1 mol L^− 1^ diluted hydrochloric acid was added, and the tube was continuously shaken for 12 h for extraction. The liquid was then filtered, and the concentration of iron in the supernatant was determined by inductively coupled plasma-optical emission spectrometry (ICP-OES; PE Optima 8000DV, USA) [32].

### Determination of total nitrogen

The samples were heated at 105 °C for 30 min, and then dried to constant weight at 80 °C. After being crushed through a 0.25 mm sieve, total nitrogen was determined using a Kjeltec apparatus (FOSS Kjeltec™ 8000, Denmark) [33].

### Determination of FCR activity

FCR activity was quantified by the dipyridine method. First, 0.5 g of sample was soaked in 0.5 mmol L^− 1^ CaSO_4_ for 5 minutes, washed with deionized water, and then soaked for 2 hours in 50 mL determination solution (pH 5.8) containing 0.4 mmol L^− 1^ dipyridine and 0.1 mmol L^− 1^ FeNa-EDTA. The absorbance of the solution was determined by spectrophotometer (Shimadzu UV 1800, Japan) at 523.3 nm [34].

### Determination of NO content

Nitric oxide content was determined according to the method of nitrate reductase, which is used to specifically reduce NO_3_^−^ to NO_2_^−^. Through color development, colorimetry was performed at the wavelength of 550 nm and light diameter of 0.5 cm. A NO determination kit was used to measure the amount of NO (JianCheng A0112–1, Nanjing, China).

### Determination of nitrogen assimilation-related enzymatic activities

Nitrate reductase (NR), nitrite reductase (NiR), glutamine synthetase (GS), glutamate synthetase (GOGAT), and glutamate dehydrogenase (GDH) were determined using activity detection kits (SolarBio, Beijing, China). NR activity was determined by soaking in inducer for 2 h, drying by filter paper, and freezing at − 20 °C for 30 min. After the filter paper was aspirated to dryness, 0.1 g sample was weighed, and 1 mL extract was added. The sample was ground in an ice bath, centrifuged at 4000 rpm and 4 °C for 10 min, and the supernatant was placed on ice for testing. NiR and GOGAT activity was determined by weighing 0.1 g sample, adding 1 mL extract, grinding in an ice bath, centrifuging at 10,000 rpm and 4 °C for 10 min, and then, the supernatant was removed and placed on ice for testing.

For determination of the GS and GDH activity, 0.1 g sample was weighed, and 1 mL extract was added. The sample was ground in ice bath, centrifuged at 8000 rpm and 4 °C for 10 min, and the supernatant was then removed and placed on ice for testing. Deionized water was used as the reference solution. The colorimetric wavelengths of NR, GDH, and GOGAT are 340 nm, while those of NiR and GS are 540 nm.

### Real-time quantitative PCR (RT-qPCR) analysis

The homologous sequence of pear was found in the National Center for Biotechnology Information (NCBI) database, and the full length of the pear genome was obtained by comparison. RT-qPCR primers were designed (Table 1), and the primers were synthesized by Beijing Tsingke Biotechnology Co., Ltd. The total RNA of pear leaves that underwent different treatments was extracted using an EASY Spin Plant RNA Kit (Tiangen Biotech Co., China). cDNA was synthesized using a reverse transcription kit (Vazyme Biotech Co., China), and a Real-time quantitative PCR System (Lightcycler® 480II System, Roche) and ChamQ SYBR Color qPCR Master Kit (Vazyme Biotech Co., China) were used to analyze the relative gene expression levels under different treatments. The reaction system (20 μL total volume) consisted of template cDNA 2 μL, forward primer and reverse primer 1 μL each, Supermix 10 μL, and RNA-free H_2_O 6 μL. The reaction process was as follows: 95 °C for 5 min, 45 cycles at 95 °C for 15 s, 60 °C for 30 s, and 72 °C for 30 s [35]. Using *PbActin1* and *PbActin2* as internal reference genes, the relative expression levels for each gene were calculated by the 2^−ΔΔCT^ method, and each sample analysis was repeated 3 times [36].

**Table 1:** List of RT-qPCR Primers

Gene	Forward (5′-3′)	Reverse (5′-3′)
*PbActin1*	ACAGTGTCTGGATTGGAGGGTC	CATTTGGAGAACTCAGAAGCAC
*PbActin2*	CCTTCAATGTGCCTGCTATGTATGT	CCAGCAAGGTCCAGACGAAGAAT
*PbNR*	TACTGGTGCTGGTGTTTCTG	TCATTCCCATGACGTTCC
*PbNiR*	CGGAAGTTGGATTGACG	GCAAATTGAATCCGAACC
*PbGS*	AGCAAAGCAAGGACTCTGCC	AGCCCTCTTGTTGGTTGGAA
*PbGOGAT*	GCTGTTCAAAATGGTTGCCA	CAGCACCAACAGCAAGAAGG
*PbGDH*	CCTGAGCGCATGCTTGTTTT	AAACCGGAAACCACCCCTAC
*PbFRO2*	CCGGTCTTTCGTCGAGTCAA	TGGGCCACTAACAAGAACCC
*PbFRO4*	CTCCACAAGACGTCCGGTAA	TGGCAAGAGACCAGACCAAG
*PbIRT1*	GCTGAAGCATCGTGTTGTCG	AAATGGCAGCAATGAGTGGC
*PbIRT1-like*	GCAGCCATTGCTATGGGGAT	CAATGCCGATGCCTACTCCA

### Statistical analysis

All data were processed using DPS and GraphPad Prism 6 software, and are presented as the mean ± standard error (mean ± SD) of triplicate determinations. Differences were defined as significant at *P*<0.05 (least significant difference (LSD) test). All experiments were conducted at least in triplicate.

## Results

### Effects of exogenous NH_4_NO_3_ and NO treatment on the growth and development of pear under iron deficiency

**Fig. 1:**
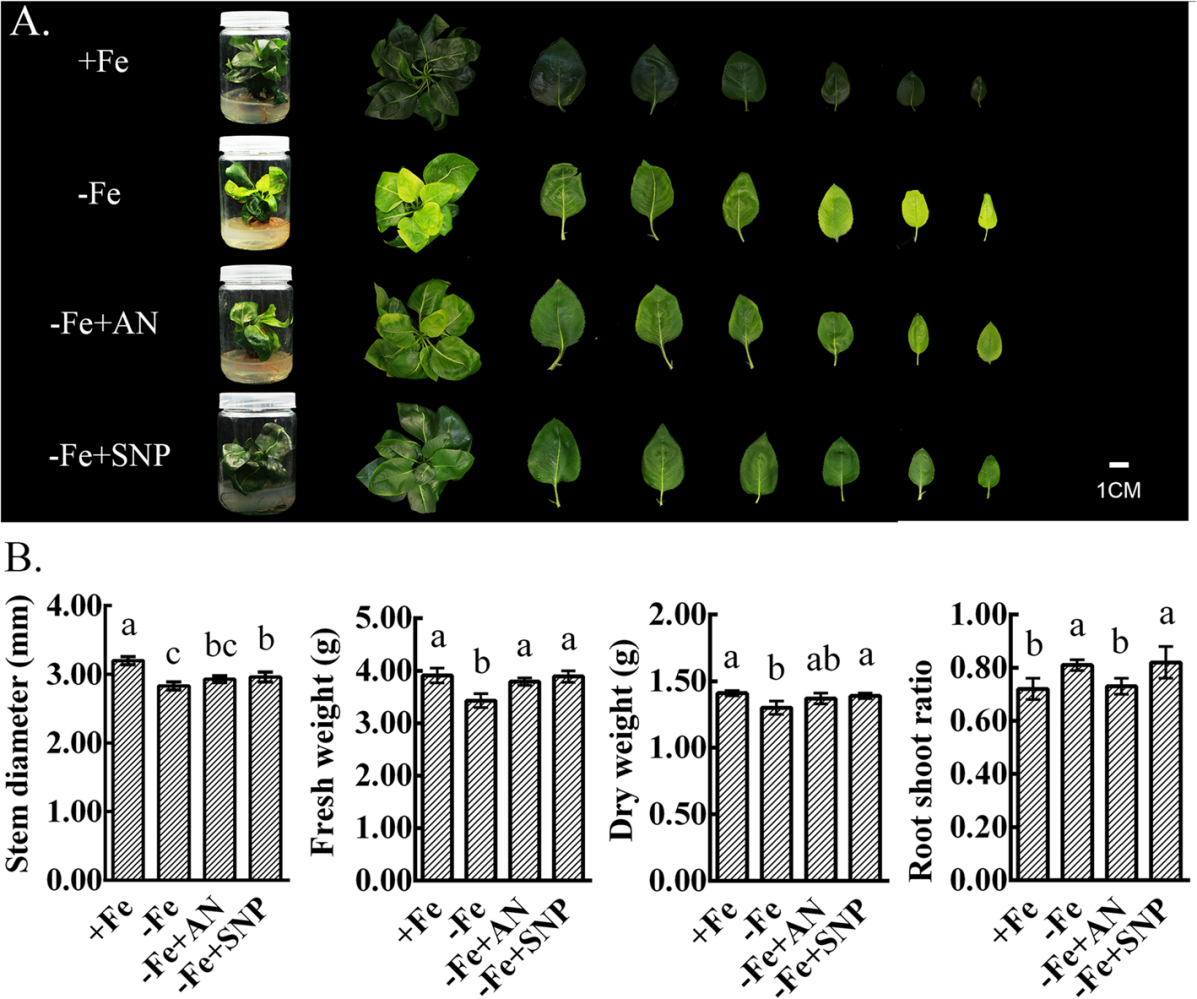
Effects of exogenous NH_4_NO_3_ and NO application on the growth and development of pear with iron deficiency. **A** The growth phenotypes. **B** Growth index, including stem diameter, fresh weight, dry weight, and root shoot ratio. The data are expressed as the mean ± SD (*n* = 6). Different letters indicate significant differences at *P* < 0.05 (LSD test)

Exogenous NH_4_NO_3_ and NO effectively alleviated the chlorosis of new leaves by iron deficiency stress, with NO exerting a stronger effect (Fig. 1A). Iron deficiency significantly decreased stem diameter and fresh and dry weights, and inhibited the overall growth of plants. NH_4_NO_3_ and NO partially restored plant growth and increased stem diameter and fresh and dry weights. Under iron deficiency, the root-shoot ratio of plants increased, indicating that iron deficiency had a greater inhibitory effect on shoots than roots. Exogenous NH_4_NO_3_ reduced the root-shoot ratio, but exogenous NO had no effect on the root-shoot ratio (Fig. 1B).

### Effects of exogenous NH_4_NO_3_ and NO on the relative chlorophyll content and photosynthetic capacity of pear leaves under iron deficiency

**Fig. 2:**
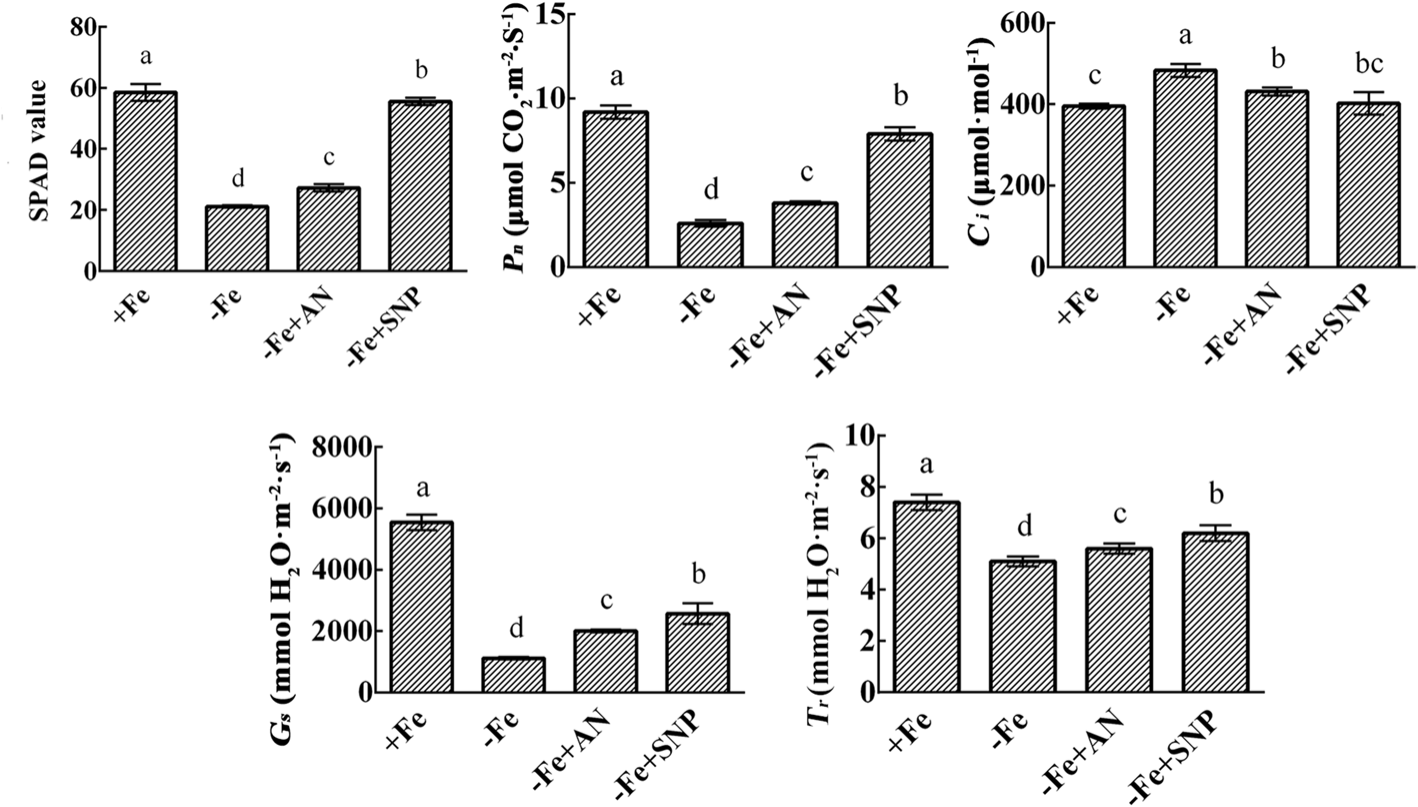
Effects of exogenous NH_4_NO_3_ and NO on SPAD value and photosynthetic performance of iron-deficient pear leaves. The data are expressed as the mean ± SD (*n* = 6). Different letters indicate significant differences at *P* < 0.05 (LSD test)

Iron deficiency decreased the SPAD value and the photosynthetic capacity of pears. Exogenous NH_4_NO_3_ and NO significantly increased the SPAD value and the net photosynthesis rate of pear leaves under iron deficiency. In addition, Fe deficiency significantly decreased the stomatal conductance and transpiration rate, and increased the intercellular CO_2_ concentration. Under Fe deficiency, exogenous NH_4_NO_3_ and NO significantly increased the stomatal conductance and transpiration rate, and decreased the intercellular CO_2_ concentration of mesophyll cells (Fig. 2).

### Effects of exogenous NH_4_NO_3_ and NO on the root growth of pear under iron deficiency

**Fig. 3:**
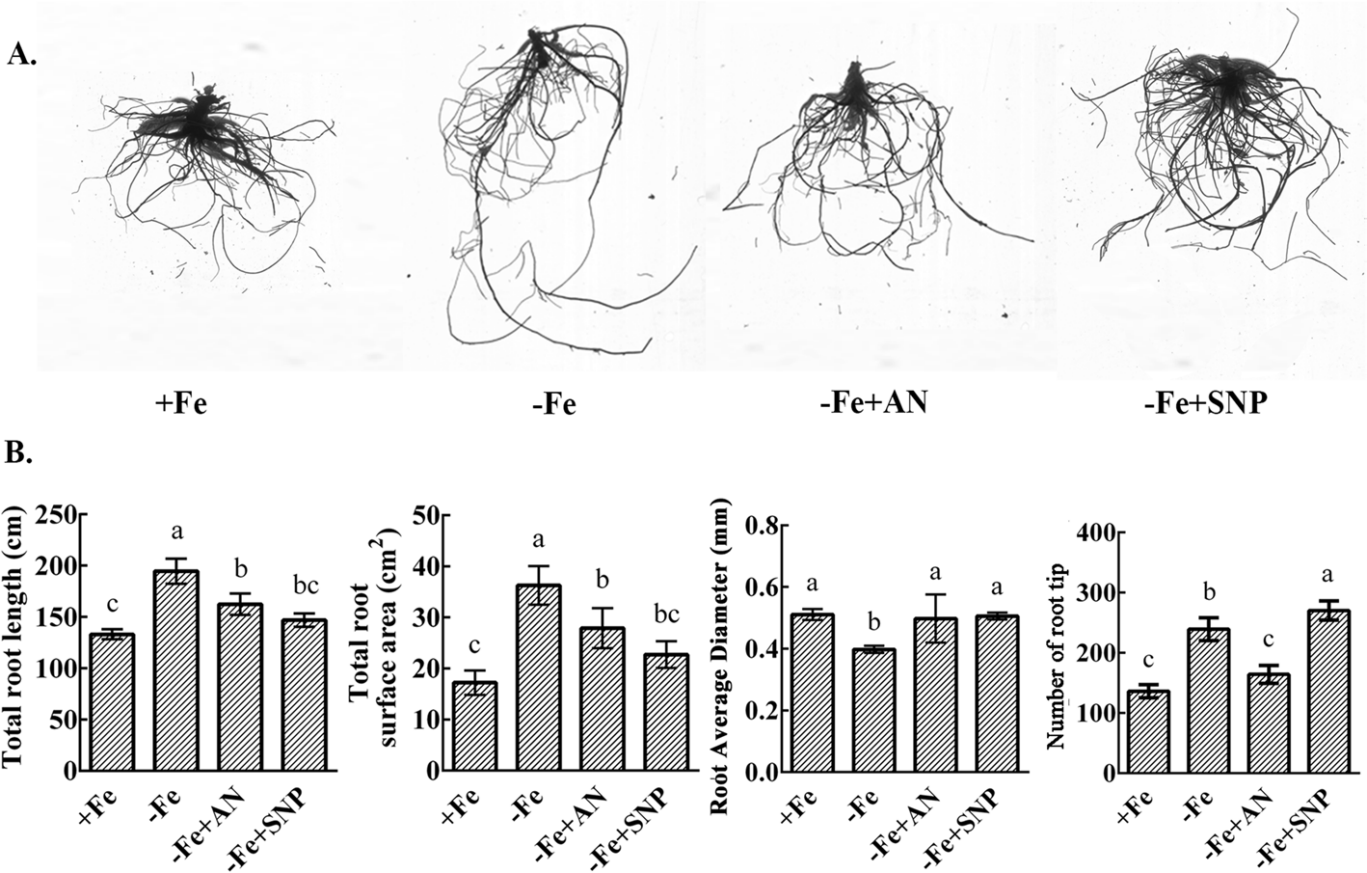
Effects of exogenous NH_4_NO_3_ and NO on the root phenotype of pear under iron deficiency stress. **A** Root phenotype of pear treated with NH_4_NO_3_ and NO. **B** Effects of exogenous NH_4_NO_3_ and NO on root length, root surface area, root average diameter, and total root tip number under iron deficiency. The data are expressed as the mean ± SD (*n* = 3). Different letters indicate significant differences at *P* < 0.05 (LSD test)

As the most important organ for plant nutrient absorption, roots play an important role in the absorption and utilization of plant shoots. Iron deficiency promotes root growth (Fig. 3A). Iron deficiency increased the total root length, root surface area, and total root tip number in pears. However, iron deficiency results in a decrease in the mean root diameter. Under iron deficiency, exogenous NH_4_NO_3_ and NO significantly decreased the total root length and total root surface area of plants, but significantly increased the average root diameter. The addition of NH_4_NO_3_ significantly decreased the number of root tips, while the addition of NO significantly increased the number of root tips (Fig. 3B).

### Effects of exogenous NH_4_NO_3_ and NO on the iron absorption capacity of pear under iron deficiency

**Fig. 4:**
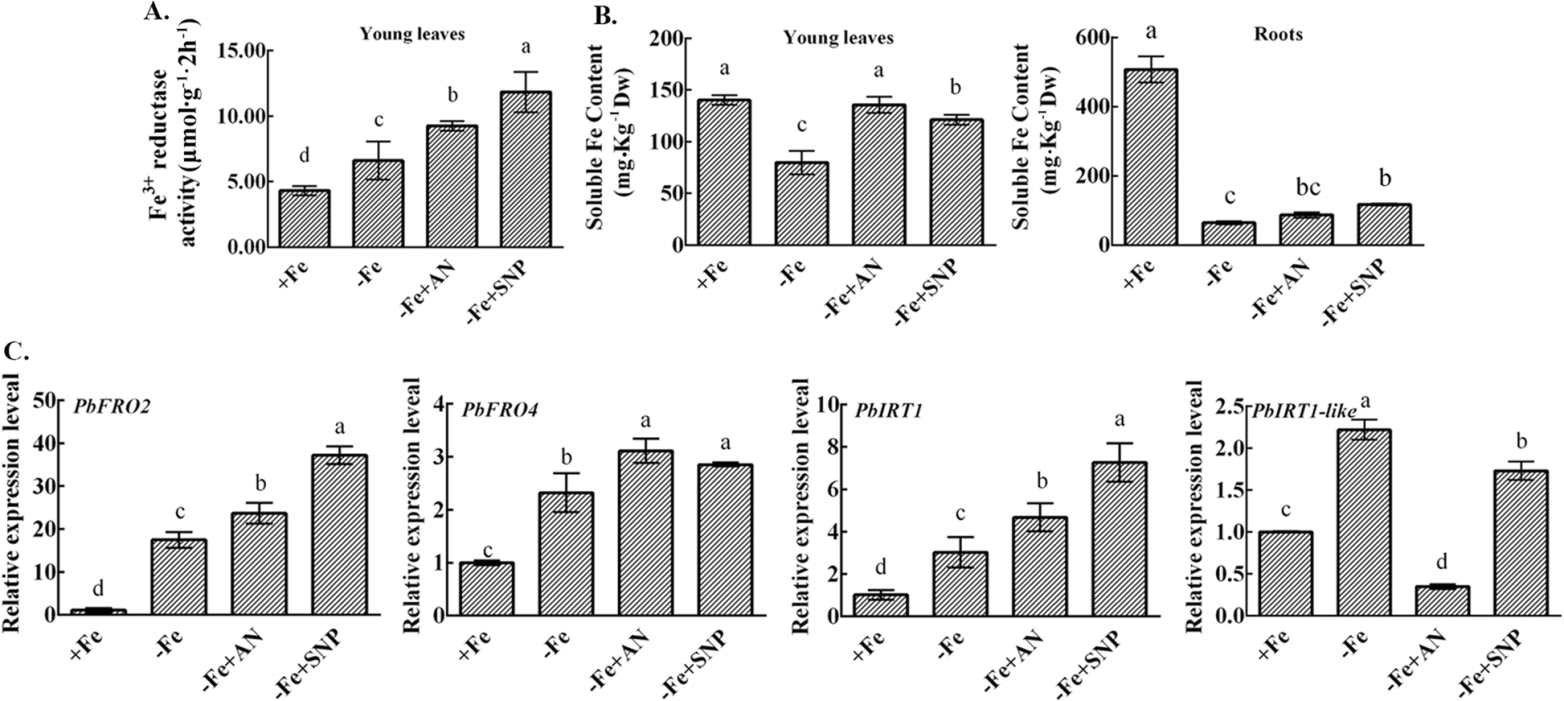
Effects of NH_4_NO_3_ and NO on iron absorption capacity and soluble iron content of pear under iron deficiency stress. **A** Effects of NH_4_NO_3_ and NO on FCR activity in pear leaves under iron deficiency. **B** Soluble Fe content of newly expanded leaves and roots. **C** The relative expression levels of iron absorption genes (*PbFRO2, PbFRO4*) and iron transport genes (*PbIRT1, PbIRT1-like*). The data are expressed as the mean ± SD (*n* = 3). Different letters indicate significant differences at *P* < 0.05 (LSD test)

To prove whether NH_4_NO_3_ and NO increased the soluble iron content in plants to alleviate iron deficiency stress, we measured the active iron content in new leaves and roots. Under iron deficiency, NH_4_NO_3_ and NO significantly increased the FCR activity (Fig. 4A) and the soluble iron content in new leaves (Fig. 4B). However, NH_4_NO_3_ did not significantly increase the soluble iron content in roots. In addition, NH_4_NO_3_ and NO increased the expression of iron absorption genes *PbFRO2* and *PbFRO4*, as well as the expression of iron transport-related genes *PbIRT1* (Fig. 4C). NO also significantly increased the expression of *PbIRT1-like*.

### Effects of exogenous NH_4_NO_3_ and NO on the total nitrogen content and NO content in pear under iron deficiency

**Fig. 5:**
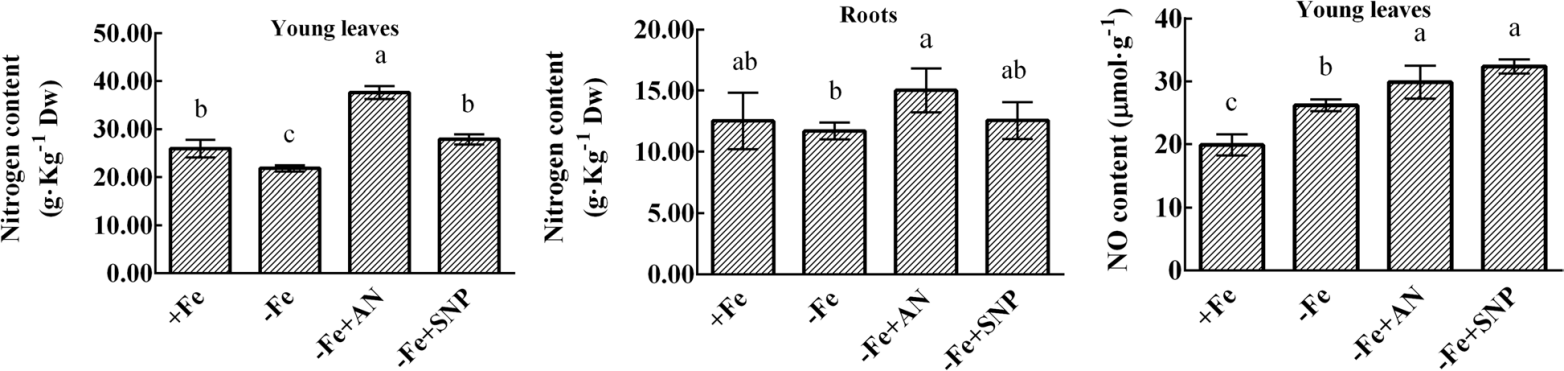
Effects of exogenous NH_4_NO_3_ and NO on total nitrogen content and NO content in pear under iron deficiency. The data are expressed as the mean ± SD (*n* = 6). Different letters indicate significant differences at *P* < 0.05 (LSD test)

Iron deficiency significantly decreased the total nitrogen content in roots. Under iron deficiency, exogenous NH_4_NO_3_ significantly increased the total nitrogen content in young leaves and roots. Exogenous NO also significantly increased the total nitrogen content in young leaves, but had NO significant effect on the total nitrogen content in roots. Iron deficiency significantly increased the NO in young leaves, and exogenous NH_4_NO_3_ and NO increased the NO content in young leaves under iron deficiency (Fig. 5).

### NH_4_NO_3_ alleviates leaf chlorosis through NO

**Fig. 6:**
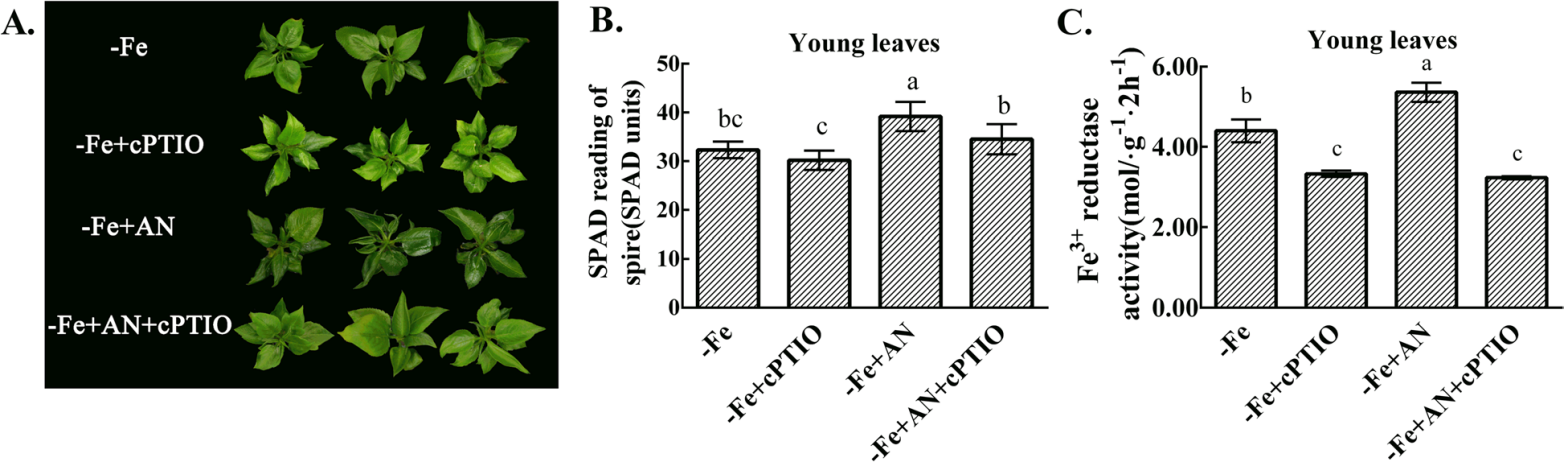
Effects of exogenous NH_4_NO_3_ and cPTIO application on SPAD value and FCR activity of pear with iron deficiency. **A** The growth phenotypes. **B** SPAD value. **C** The FCR activity data are expressed as the mean ± SD (*n* = 6). Different letters indicate significant differences at *P* < 0.05 (LSD test)

The chlorosis of leaves were accelerated by cPTIO treatment under iron deficiency. Ammonium nitrate treatment alleviated leaf chlorosis, and the SPAD value was significantly higher than that under iron deficiency. However, the chlorosis of leaves were not alleviated after treatment with ammonium nitrate and cPTIO (Fig. 6). cPTIO treatment significantly decreased the activity of Fe^3+^ reductase. Ammonium nitrate treatment increased the activity of Fe^3+^ reductase, but when the treatment with ammonium nitrate and cPTIO, the activity of Fe^3+^ reductase did not increase and was significantly lower than that under iron deficiency (Fig. 6C).

### Exogenous NH_4_NO_3_ and NO increased the activities of nitrogen assimilation-related enzymes in pear leaves under iron deficiency

**Fig. 7:**
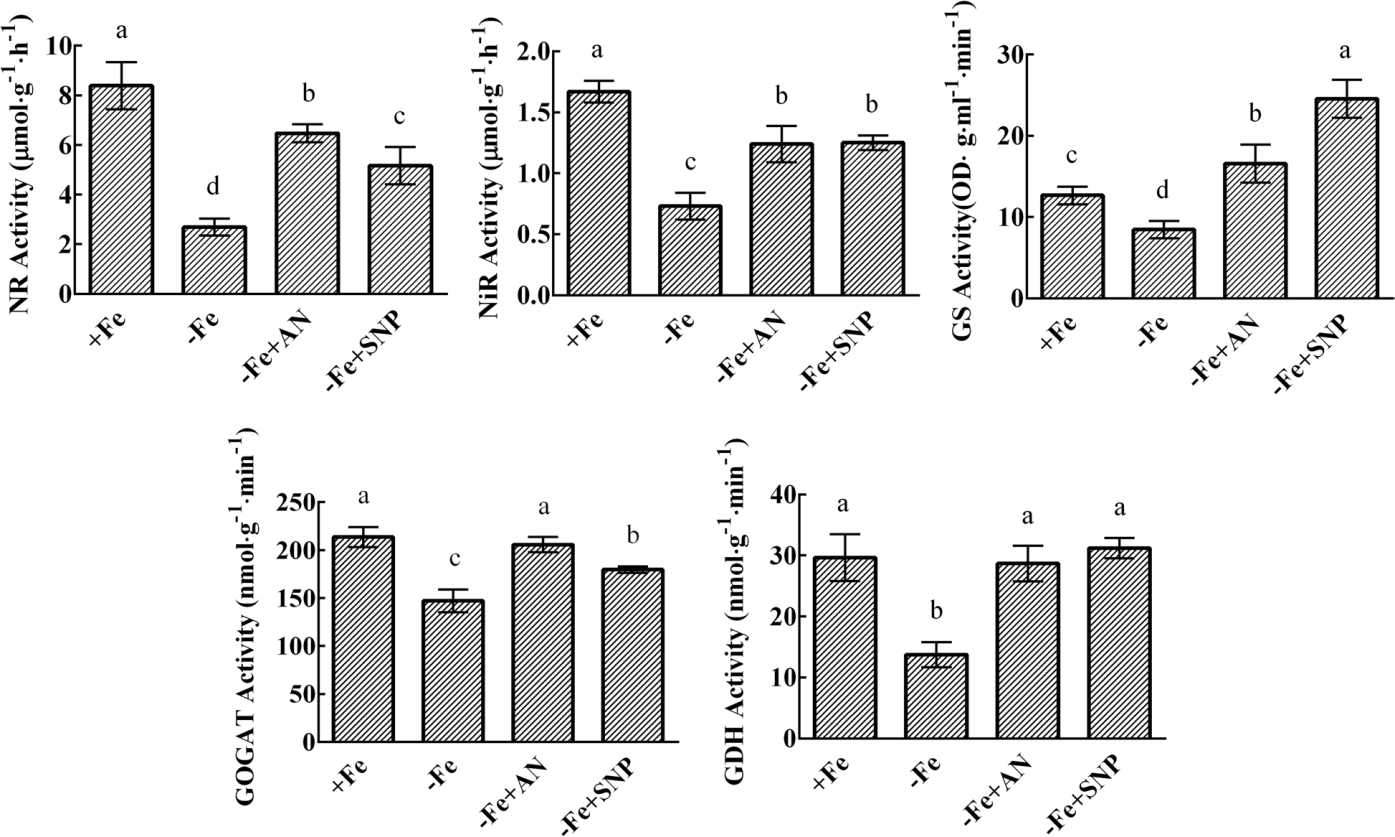
Effects of exogenous NH_4_NO_3_ and NO on nitrogen metabolism-related enzymatic activities. The data are expressed as the mean ± SD (*n* = 3). Different letters indicate significant differences at *P* < 0.05 (LSD test)

**Fig. 8:**
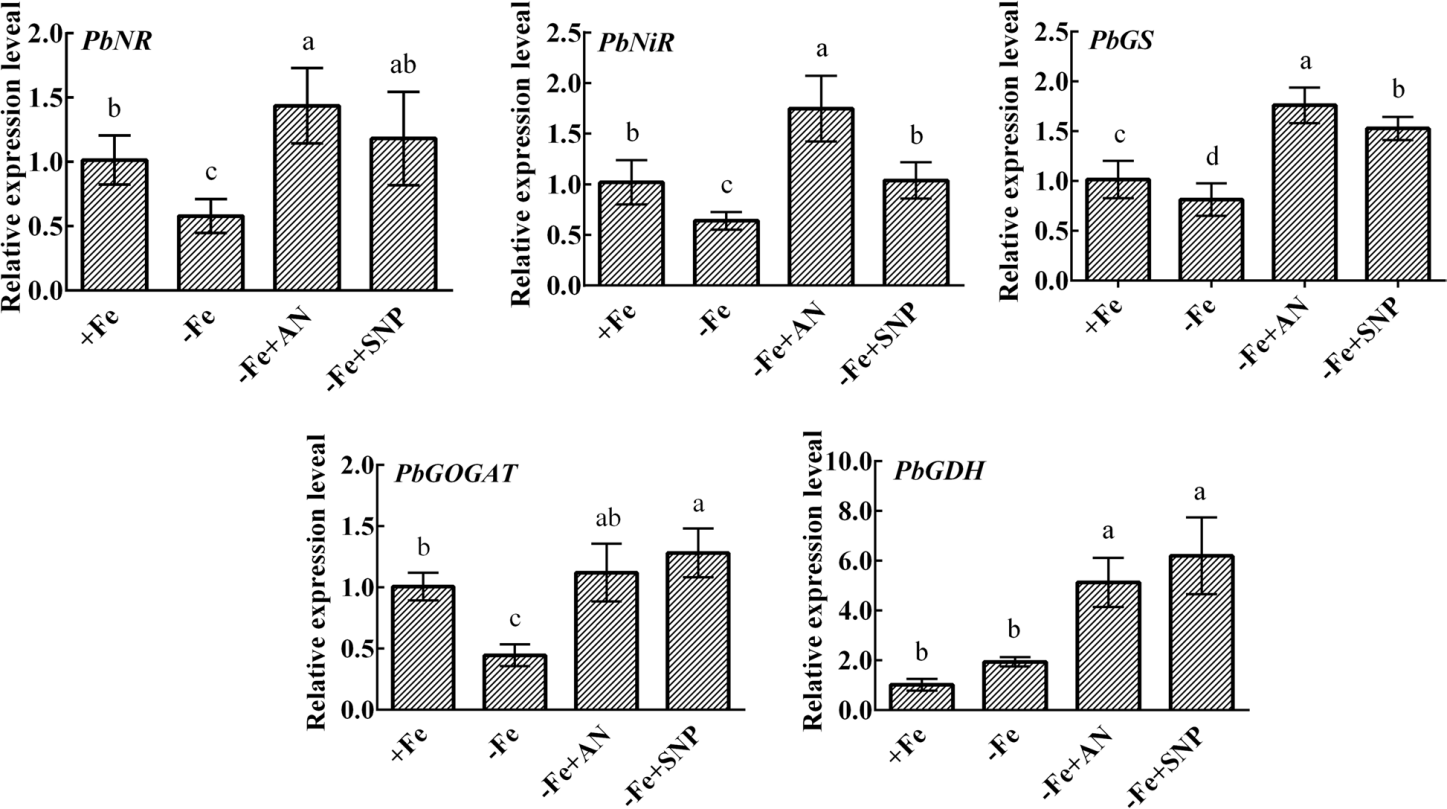
Effects of exogenous NH_4_NO_3_ and NO on relative expression levels of genes related to nitrogen metabolism. The data are expressed as the mean ± SD (*n* = 3). Different letters indicate significant differences at *P* < 0.05 (LSD test)

Iron deficiency decreased the activities of NR, NiR, GS, GOGAT, and GDH, while exogenous NH_4_NO_3_ and NO significantly increased these enzymatic activities under iron deficiency. After treatment with NH_4_NO_3_, the activities of NR and NiR were significantly increased, but did not reach normal levels. Exogenous NH_4_NO_3_ and NO significantly increased the activity of GS to levels even higher than those of the normal iron treatment group. Compared with NO treatment, GOGAT activity and NiR activity were significantly increased after ammonium nitrate treatment (Fig. 7). NH_4_NO_3_ and NO significantly increased the relative expression levels of nitrogen assimilation-related genes *PbNR*, *PbNiR*, *PbGS*, *PbGOGAT*, and *PbGDH* (Fig. 8).

## Discussion

Fruit trees are very sensitive to iron deficiency. Pear is one of the species of iron deficiency-sensitive fruit trees. Under iron deficiency stress, the young leaves at the tip of the new shoot lose their green color first. With the aggravation of stress, the mesophyll gradually loses its green color and turns yellow. In severe cases, the green color of the leaf veins fades, the entire leaf turns yellowish white, and dry patches occur at the edge of the leaves. Finally, the leaves dry and fall off, which eventually leads to a decrease in tree vigor and fruit quality, and even death, causing great economic losses to growers [37, 38].

In the current study, under iron deficiency, the chlorophyll content of pear variety ‘Qingzhen D1’ decreased. The leaves from young to old gradually turned yellow, resulting in the yellowing phenotype of the plant, which inhibited photosynthesis and finally led to thinning of the stem and poor growth. After NO treatment, we found that the phenomenon of plant yellowing was alleviated, and plant growth did not cause significant changes at 60 days. Previous studies have reported the role of NO in plants under iron deficiency stress. It is considered that the accumulation of NO caused by iron deficiency is a factor in the response to iron deficiency stress. NO alleviates iron deficiency stress in plants through increasing FCR activity and improving iron ion transport [39, 40]. This is consistent with our research results. As an important nitrogen source for plants, we used ammonium nitrate to treat plants under iron deficiency stress. It was found that its effect of alleviating iron deficiency stress was weaker than that of NO, but it also significantly increased plant growth and alleviated plant yellowing compared with the iron deficiency treatment group.

Through observation of root growth, we found that iron deficiency resulted in the growth of roots. In contrast, the treatment of ammonium nitrate and NO weakened this phenotype, and root growth was inhibited, but the root length and root surface area were significantly higher than those of plants that received a normal iron treatment. It is worth noting that the number of root tips in the no-treatment group significantly increased. In previous studies, NO promoted root tip growth, and it may act in different pathways with root growth caused by iron deficiency [41, 42].

To further understand the effects of ammonium nitrate and NO on plant iron absorption, we measured the soluble iron content and FCR activity. Under iron deficiency, ammonium nitrate and NO treatment significantly increased the activity of FCR. The soluble iron content significantly increased in the underground and aboveground parts of the plant. In pear (a strategy I plant), free iron is reduced to Fe^2+^ by FCR on the plasma membrane, and it is then transported to the cell through *IRT1*. qPCR analysis showed that ammonium nitrate and NO promoted the high expression of *PbIRT1* and *PbFRO1*. For the *Arabidopsis* homologous *PbIRT1* gene [43], NO significantly increased its expression, while ammonium nitrate did not result in a high expression trend. This shows that both ammonium nitrate and NO increase the absorption of irons, and there are more transporter proteins for iron regulated by NO than those ammonium nitrate, which may be the reason for the difference between them in alleviating plant iron deficiency.

Iron deficiency stress decreased nitrogen accumulation, especially aboveground nitrogen accumulation. Ammonium nitrate as a nitrogen source induced nitrogen accumulation. Interestingly, NO treatment also increased nitrogen accumulation. Meanwhile, we found NO increased after ammonium nitrate treatment, which may indicate that ammonium nitrate could indirectly affect the response to iron deficiency through NO. Ammonium nitrate could alleviate the chlorosis caused by iron deficiency, but the alleviating effect was weakened after adding cPTIO. Additionally, leaf chlorosis was more severe than iron deficiency, which indicated that NO is important for facilitating the action of ammonium nitrate in alleviating leaf chlorosis under iron deficiency. The nitrogen cycle also plays an important role in alleviating iron deficiency. Iron deficiency significantly decreased the nitrogen metabolism level in *Areca catechu* L., and similar conclusions were also reached in cucumber and *Arabidopsis* [12, 44, 45], which is also consistent with our study.

Through the determination of key enzymatic activities and the identification of related gene expression, it was found that the activities of NR, NiR, GS, GOGAT, and GDH were significantly increased after treatment, and the activity of GS after treatment was higher than that in the normal Fe application group. This may be due to the increase in NH_4_^+^ caused by exogenous ammonium nitrate and NO treatment in plants. GS has high affinity for NH_4_^+^ [46], which can ensure that the plant maintains a low level of NH_4_^+^ to protect its tissue from damage.

## Conclusions

**Fig. 9:**
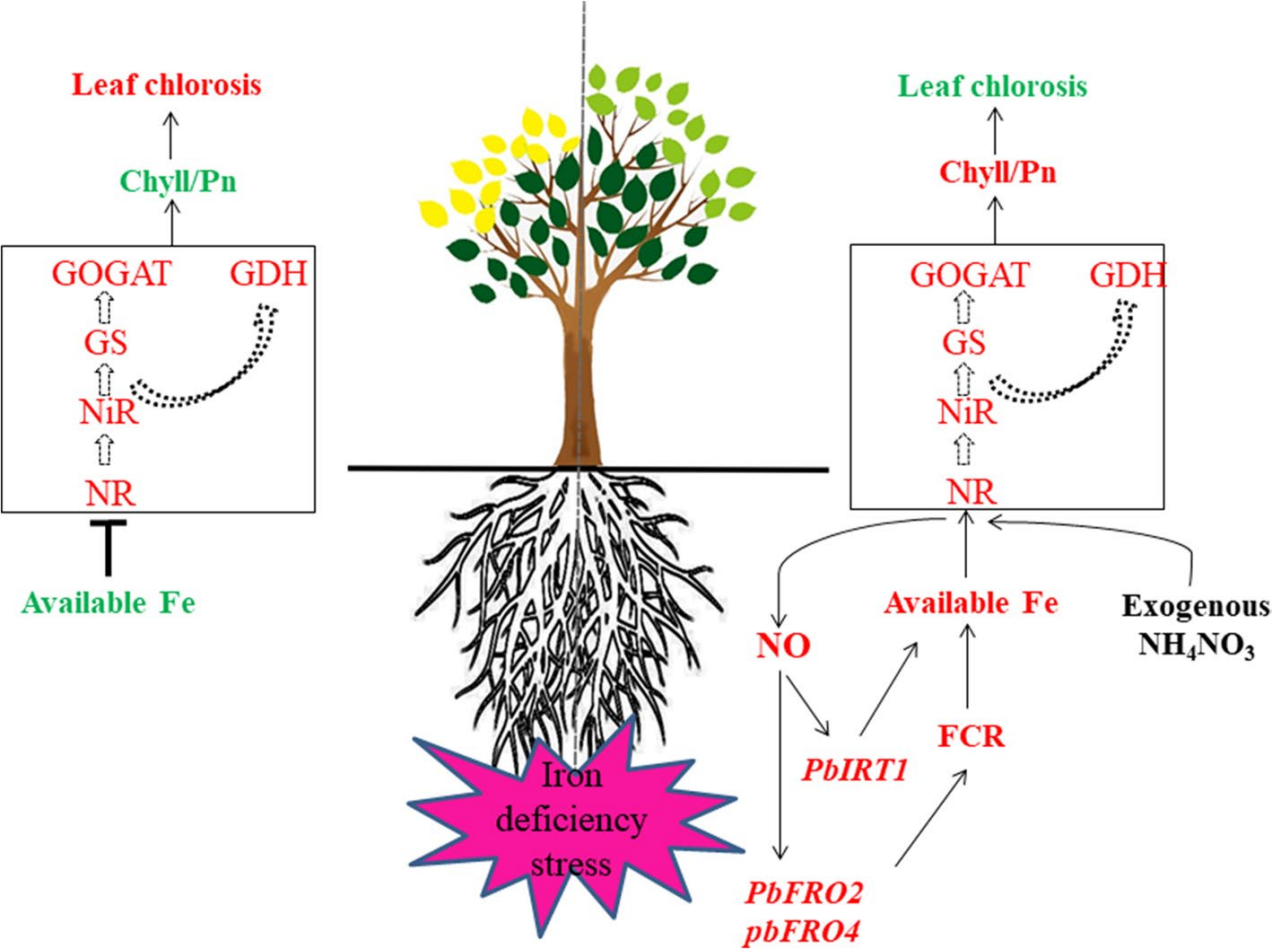
A schematic model for alleviating iron deficiency chlorosis in pear mediated by NH_4_NO_3_ and NO. Red text indicates promotion, and green text indicates inhibition

We used ammonium nitrate and NO treatment to explore the effects of the two substances on iron deficiency stress. It was found that NO promoted plant nitrogen accumulation and alleviated the yellowing symptoms caused by iron deficiency. Ammonium nitrate can indirectly alleviate the symptoms of plant yellowing by promoting the increase of NO, but it is not the only method. NO increases the response of iron transporters. Both substances increase nitrogen accumulation in plants by promoting the activity of key enzymes of nitrogen assimilation. The increase in GS activity alleviated the adverse effects of excessive NH_4_^+^ to a certain extent (Fig. 9). This study provides a theoretical basis for further examination of iron stress alleviation in plants.

## Data Availability

All data generated or analyzed during this study are included in this published article.
